# Perspectives of Translational Medicine

**Published:** 2015

**Authors:** Lingqun Hu, John Zhang, Weifeng Tu, Renyu Liu, Li-Ming Zhang

**Affiliations:** 1 Department of Anesthesiology, Northwestern University; 2 Departments of Neurosurgery, Loma Linda University School of Medicine; 3 Department of Anesthesiology, Guangzhou General Hospital of Guangzhou Military Command, Guangzhou, China; 4 Department of Anesthesiology and Critical Care, University of Pennsylvania; 5 Department of Anesthesiology, University of Pittsburgh School of Medicine

Recently, we had a well-moderated, multidisciplinary discussion on perspectives of translational medicine including experts from both the United States and China through the WeChat group for Translational Perioperative and Pain Medicine (TPPM) hosted by Dr Renyu Liu, the interim Editor-in-Chief of TPPM. This proved to be an effective approach in promoting international academic exchanges and discussions using social media in an organized manner. The discussions are included below with minimal editing to improve clarity.

## John Zhang (Editor in Chief, Translational Stroke Research, Loma Linda University Medical Center, USA)

Dr. Renyu Liu asked to discuss perspectives on translational medicine and research. Here I simply make the following two points and hope for your enthusiastic participation in the discussions. Firstly, translational medicine has become a very hot topic; the United States Governmental agencies, including the United States National Institutes of Health (NIH) and many colleges and universities are all promoting translational medicine. This has affected the grant review process and grant funding directions, where grant proposals without significant translational components tend to have less funding opportunities. The grant funding trend in China is similar to that in the United States. On the other hand, many experts believe basic scientific research should be strengthened further because it currently overemphasizes the importance of translational medicine. In my opinion, basic science and translational research should develop in parallel, since translational medicine emphasizes the transition of basic science towards clinical application, most importantly, to transform preclinical experiments into clinical trials. In Europe, for example, stroke researchers are exploring the establishment of an Experimental Center to launch comprehensive and systematic studies to speed up such transition.

I am collaborating with Chinese colleagues to carry out a similar task. In the past two years, I have been collaborating with Beijing Xuanwu Hospital to set up a Trial Center. Although we’ve done a lot of preliminary work, we are still actively pursuing sufficient funding for the Trial Center.

## Weifeng Tu (Military General Hospital of Guang-zhou, China)

Translational medicine has much operational value. The extensive attention of grant funding organizations and researchers to translational medicine also reflects that research in basic sciences might have deviated from its final goals (short, middle or long-term). This may have occurred. Many basic science studies cannot produce any clinical value due to the nature of the research itself; being rather for the sake of obtaining knowledge, receiving funding, or publication with Science Citation Index (SCI).

## John Zhang (USA)

Your perspective is similar to that which the majority of people hold. However, do you think translational medicine will be successful if one lacks understanding of basic sciences? Moreover, the majority of research in translational medicine has failed. Do you think it is time to strengthen research and understanding of basic sciences?

## Hailong Dong (Xijing Hospital, China)

Regardless, we should still perform large-scaled and clinically relevant research in translational medicine. Let’s work hard to achieve this goal.

## Weifeng Tu (China)

Translational medicine should act as a bridge between basic scientific research and clinical practice needs/issues. It can increase our ability to communicate, to exchange, and to understand. In order to increase the efficiency of translational medicine, clinicians should raise clinical questions and demands based upon the findings of basic scientific research. Both research in basic science and translational medicine should depend upon each other. Otherwise, we will return to the original problem of placing too much emphasis on the application of research.

## John Zhang (USA)

This is an opinion from an opposing viewpoint. Should we divide funding equally between both basic and translational research?

## Renyu Liu (University of Pennsylvania, USA)

These two issues raised by Dr. John Zhang are very important for a valuable discussion. The National Institute of Health (NIH) has fully recognized the importance of translational medicine and has established special funding for this purpose. Moreover, NIH provides available resources to assist scientific researchers (both in basic and clinical) to develop translational medicine. Many large scientific research organizations, as well as colleges and universities, have invested huge amounts of money to establish Translational Medical Centers. Translational medicine is speeding up as well in China.

There are many problems with transforming basic scientific research into clinical application. There may be due to less attention shown towards practical research or basic science research headed the wrong direction. Perhaps they stem from too much mechanistic research hindering the development of translational medicine. Maybe people too often dismiss unpatented scientific findings, simply cannot find appropriate funding, or fail to create a suitable developmental or a business plan? Stroke research provides a good example. Searching on PubMed (www.pubmed.org), one can find 222,443 stroke-related publications in Feb 2015; however, there is very limited therapeutics for stroke in humans except tissue plasminogen activator.

## Xuefeng Ling (Stanford University, USA)

Translational science research is very difficult to get funding for because the NIH wants to promote deep exploration for mechanisms. If your research doesn’t have that, in the case of applied science research, it is difficult to get grants.

## Renyu Liu (USA)

It is true that translational and applied sciences are historically difficult to get funding. The tides are changing gradually, as pointed by Dr. John Zhang.

## Lingqun Hu (Northwestern University, USA)

True. However, there is no complete system.

## Weifeng Wu (China)

I personally feel translational medicine currently seems a little bit superficial.

## Renyu Liu (USA)

One of my colleagues said to me that translational medicine is a joke, and at present, deviates too far from the goal of achieving a better outcome for patients. Translational medicine should focus on patient needs. That is the true essence of medicine. I would say translational medicine did not deviate from its goal by itself, but many people use the term “translational medicine” in a misleading way.

## Weifeng Tu (China)

I agree with you.

## Juan Li (Anhui Provincial Hospital, China)

That’s right. It surely is.

## Lingqun Hu (USA)

I would think so too.

## Xue Bai (Chinese Society of Anesthesiology, China)

This is just like what people said about money being evil. However, it is not money which is evil, but the greed which it inspires.

## Henry Liu (Tulane University, USA)

Without any doubt, translational medicine is a very hot topic nowadays. It is understandable because the ultimate goal of any kind of biomedical research is to serve our patients. In Western countries, the process from “Bench to Bedside” is relatively quick due to enormous commercial interest after the successful clinical translation. I remember reading some of the pioneer research in transcutaneous oxygenation saturation measurement in the mid 80’s. By the time I started my clinical practice in the United States in the early 90’s, and pulse oximetry (SpO2) was already widely used in the clinical settings in the United States.

In China, it seems to me that there could be advantageous in clinical research in developing new drugs and new technologies, and translational medicine, because China consists of almost ethnically homogenous population, large samples can easily be obtained in the well-controlled condition and in a relatively short period of time for a particular procedure (even in a single hospital). For example, I recently learned that Dr. Shihai Zhang at Wuhan Union Hospital was able to collect 500 cases of mitral valve replacement (MVR) for a well-designed study in short 4 months. This is extremely hard to achieve in any single medical center in the United States. When conducting a multi-center clinical trial, it is advantageous if you can get huge sample size in a relatively shorter period of time. I would strongly suggest to our colleagues in the Chinese Society of Anesthesiology to take full use of these advantages.

There is an increasing trend of extensive collaborations between academicians and pharmaceutical companies. Such collaborations between scientists and product development companies will undoubtedly shorten the transforming time from laboratory to clinical practice. When I visited the High-Tech District at Wuhan Guanggu and Shanghai Zhangjian, I noticed that many start-up biotech companies have been established in those places. I truly believe there will be a rapid development of life sciences in China for the coming decades. Our anesthesia colleagues should grasp this opportunity to make the anesthesiology specialty keeping up with the international progress in science and technology, especially in developing new equipments and methodologies. I appreciate Dr. Renyu Liu at the University of Pennsylvania who established this WeChat group to promote translational medicine in Perioperative and Pain Medicine.

## Weifeng Yu (President of Chinese Association of Anesthesiologist, Renji Hospital, China)

I greatly admire the academician Gu Yudong’s clinical scientific research principles: 1+X> S. In this equation, the “1” means everything is for the patient interest, to provide the first-class quality of service. The “+” represents the goals of scientific research: the addition of knowledge and experiences in addition to innovations. The “x” is the logic of scientific research with your questions and problems, and the “S” is the attitude of scientific research emphasizing the developmental process rather than the final destination of success and seeking for team spirit rather than individual effort. Therefore, my research is more focused on the value of clinical application and looking forward to better outcomes in translational medicine.

## Xiangming Fang (Zhejiang University, China)

I totally agree.

## Li-Ming Zhang (University of Pittsburgh Medical Center, USA)

Professor Yu’s comments should guide scientific research, particularly for clinicians in China. Specifically, clinical studies should aim towards answering clinical questions and problems, using teamwork to organize a large-scale multi-hospital or multi-center study and fully take the advantage of large clinical samples in China. This will allow China to make more contributions to the advancement of medical sciences in the world.

## Buwei Yu (Immediate Past President of Chinese Society of Anesthesiology, Ruijin Hospital, China)

Cultivating a consciousness has a much more lasting impact than participating in a campaign does. Hopefully, translational medicine will not be seen as a hard-set rulebook similar to that for evidence-based medicine by some experts.

## Weifeng Tu (China)

Translational medicine suggests that every clinician and researcher in basic medical sciences should perform research step by step to search for real questions and problems so the research outcome could improve quality of clinical care greatly and overall well-being in patients.

## Lingzhong Meng (University of California San Francisco, USA)

Can you define translational medicine in just one sentence?

## Lei Fang (Lawyer, Troutman Sanders LLP, USA)

Quoted from the website: “Translational medicine (also referred to as translational science) is a discipline within biomedical and public health research that aims to improve the health of individuals and the community by “translating” findings into diagnostic tools, medicines, procedures, policies and education.” (http://en.wikipedia.org/wiki/Translational_medicine)

## Li-Ming Zhang (USA)

Attorney Fang, do you think the process of simulation training and research in the laboratory that transforms the training outcome into clinical practice in patients can be defined as translational medicine?

## Lei Fang (USA)

Very good question! It depends. I believe Dr. Liu would be in a better position to provide you with a more accurate and/or practical answer.

## Weifeng Tu (USA)

Simulation training and research should be considered as part of translational medicine.

## Xuefeng Ling (USA)

Translational medicine is B2B: Bench to Bedside or laboratory to clinical practice - all innovative advances. The reason why translational medicine gets a lot of attention is because related innovations can lead to intellectual properties (==$$$$) that transform the practice of medicine. Just my two cents.

## Lei Fang (USA)

This is a wonderful “two cents”, I agree (as a patent/IP lawyer).

## Li-Ming Zhang (USA)

I would consider simulation training and research in realistic settings such as in the OR, ER or ICU as methods of directly transforming findings to bedside clinical practice in a cost-effective way. Therefore, I think these should be considered as a component of translational medicine. Any comment?

## Renyu Liu (USA)

I agree! I know you had your own answer when you proposed your question.

## Xuefeng Ling (USA)

I agree with Dr. Zhang’s opinion.

## Renyu Liu (USA)

Simulation and its related technology development in medicine are all great examples of translational medicine. Many scientific technologies are developed for simulation and medical training. They are changing our practice and patient outcome despite its related cost still being very high. The simulation center in Dr. Zhang’s institution (UPMC) is one of the most successful ones in the US.

## Xiaoying Zhang (Providence Little Company of Mary Medical Center, USA)

We, the Providence medical system in California, have been using simulation training for perioperative events for three years, with good models and successful outcomes. This training includes perioperative registered nurses (preoperative, intraoperative and postoperative care unit), surgeons, operating room technicians, hospital education staff, and anesthesiologists. This program was created by Stanford Anesthesia Department.

## Li-Ming Zhang (USA)

The WISER Simulation Center under our anesthesia department at UPMC is one of the largest simulation centers in the US (except military service) http://v.youku.com/v_show/id_XMzI3MzUwNDg0.html?x. We have trained many Chinese colleagues here. China should develop simulation centers to train clinical professionals (residents, nurses, and attending physicians) to enhance the quality of patient care and patient safety. The Chinese Society of Anesthesiologists should focus on playing a leading role in simulation training and research because this is a proven, effective way to increase patient safety. I categorized simulation training as a component of translational medicine when I gave a lecture in China several years ago. Thanks for your comments and support to this concept.

## Renyu Liu (USA)

Dr. Meng, what is your answer for your own question: “What is translational medicine in one sentence”?

## Lingzhong Meng (USA)

“Translational medicine is the application of innovation and research knowledge in patient care with the aim of improving patient outcome based on qualified outcome-measuring methods.” Therapeutic clinical trial is the prototype of translational medicine. However, it centers on patients and the beneficial effects on patients, not the gain of an individual or enterprise.

## Weifeng Tu (China)

All research should have solid grounds related to clinical practice.

## Li-Ming Zhang (USA)

I concur with Dr. Meng’s answer: B2B, Bench to Bedside. Simple to understand and accurate! Any laboratory studies that could be transformed into clinical practice in a favorable outcome should be considered as translational medicine. My two cents.

## Peishan Zhao (Tuft Medical Center, USA)

Does bench work consist only of studies in the laboratory? Do clinical trials count?

## Li-Ming Zhang (USA)

Dr. Zhao, I would consider “bench” as a broad concept. Even for clinical trials, you must collect data and analyze it at your “bench” (desk). I would say any experimental approaches, including real laboratory bench studies, biomedical studies, simulation training, and clinical trials can be categorized as “bench”.

## Peishan Zhao

Thank you.

## Liang Cheng (Indiana University School of Medicine, USA)

For the Specialized Programs of Research Excellence (SPORE) program, the National Cancer Institute (NCI) defines translational research as follows: “Translational research uses knowledge of human biology to develop and test the feasibility of cancer-relevant interventions in humans and/or determine the biological basis for observations made in individuals with cancer or in populations at risk for cancer.” (http://trp.cancer.gov/) The term “interventions” is used in its broadest sense to include molecular assays, imaging techniques, drugs, biological agents, and/ or other methodologies applicable to the prevention, early detection, diagnosis, prognosis, and/or treatment of cancer.

SPORE translational research projects may involve the use of any cellular, molecular, structural, biochemical, and/or genetic experimental approaches. By this definition, SPORE projects are permitted to move not only in the forward direction, toward clinical trials and studies in areas of prevention, early detection, treatment, development of biomarkers, and population science, but also in the reverse direction, using human biospecimens, often from clinical trials, to study new phenomena, to optimize previous findings, or to develop new hypotheses based on results from human studies. All proposed SPORE projects must be translational. When we review SPORE, we reiterate that proposals must be “translational” by the NCI definition.

## Peishan Zhao (USA)

Your response brings something to mind. I remember our elementary education once advocated our learning process should be from “book to practice”, which indicates that we should use what we’ve learned from the “bench” to the practice of the “bed”. Therefore, B2B seems to be not a new concept and perhaps was proposed initially by our Chinese educators.

## Renyu Liu (USA)

While most of us agree with the definition of B2B being “bench to bedside”, there are different interpretations for the first “B”. For example, it could be interpreted as “bedside to bedside” for innovative clinical research.

It seems the simple question from Dr. Meng from UCSF, it is actually difficult to define/answer with one simple sentence, as different subspecialties might have different interpretations. However, we are missing business as an important component of translational medicine when we only talk about the B2B model. I think that translational medicine is a process to bring any innovation (created from any resources) to clinical practice via successful business intervention. IBM: Innovation~Business~Medicine.

## Linghu Hu (USA)

My understanding to translational medicine is that no matter what you do in medicine, it must impact clinical outcomes, no matter if it is a basic research, clinical research, clinical teaching, clinical management, or innovation.

## Li-Ming Zhang (USA)

I agree with Dr. Hu’s comment. The translation should produce a good clinical outcome.

## Chao He (Taikang Life Insurance, China)

I agree with Dr. Hu.

## Renyu Liu (USA)

I think every medical researcher would like the patient to have a favorable prognosis, to recover from illness, and to have improved quality of life. However, it is a long process to evaluate if the research/innovation could produce a favorable clinical outcome and it is difficult to judge whether a specific study could change the clinical outcome at the early stage of research. We have observed many promising research projects failing prematurely without reaching the stage of clinical trials and outcome evaluation. In order to shorten the process and expedite the transformation of innovation toward clinical practice, we should strengthen our collaborations and centralize our resources, particularly to facilitate collaborations between academics and enterprises.

## Lingqun Hu (USA)

Clinical outcome is the most important key. It should not be considered as translational medicine if there is no favorable or improved clinical outcomes. By reviewing some manuscripts, we noticed that a plentiful number of animal models have been adopted in a situation that could not be duplicated with clinical settings. There is no doubt there will be different results in animal studies through data analysis. However, these experiments are for publication only. The experimental results are neither standardized toxicological studies nor do they interpret clinical phenomena in any circumstance. It seems that the misuse of nomenclature for translational medicine most likely comes from these studies. It is fortunate that it is not proportional for both basic and clinical studies.

## Lingzhong Meng (USA)

I agree. No other options.

## Xiang Qian (Stanford University, president of SCAPE, USA)

I thank Professor Liu for setting up such a platform to exchange ideas about translational medicine between China and USA. It is my honor, by request of Professor Liu, to express my personal perspectives for translational medicine. Translational medicine translates research outcomes and innovative discoveries from both laboratory and clinical settings into a product with market value and further translation into clinical practice to finally fulfill the dream of B2B (bench to bedside). For the past decade, China has invested heavily in basic sciences research. I hope China will put more resources and efforts into translational and clinical medicine. The Society of Chinese American Physician Entrepreneurs (SCAPE), which I initiated, would like to endorse translational medicine and support Professor Liu’s effort on this platform. Let’s learn from each other and make progress.

## Renyu Liu (USA)

Thank you everyone for the enthusiastic discussion. While we cannot make any solid conclusion from this discussion, such activities make us think, foster international collaborations, and help us advance translational medicine. We will organize more such discussions down the road.

## Summary

This discussion is organized by Translational Peiroperative and Pain Medicine”[1] through its WeChat Group, which had 175 members on board at the time of this discussion. The members of the group include basic scientists, clinicians, physician scientists from various disciplines, hospital and medical society leaders, entrepreneurs, journal editors, attorneys, medical industry leaders, as well as professionals from other disciplines, from around the United States, Europe, and China. This is a great example of using social media as an innovative way for a multidisciplinary, international discussion on a topic related to medicine which has been widely discussed over the past few years: translational medicine.[2–5] Currently, translational medicine and Translational Medical Centers are booming in both the US and China.[6–8]

In addition, searching for the term “translational medicine” as part of the article titles on PubMed (http://www.ncbi.nlm.nih.gov/pubmed/) led us to 484 indexed papers as of February 20, 2015. The term “translational medicine” has been used in article titles dating back to 1996[9]. Multiple papers have specifically discussed the question of what translational medicine or translational research actually is[10–12]. While it seems that the importance of translational medicine is well-accepted and vigorously promoted by scientists, clinicians, physician scientists, funding agencies, policy makers, and medical industries, the true meaning of translational medicine is still debatable. There is a general consensus that improved patient outcome should be the final goal of any type of translational medicine, which includes, but is not limited to, laboratory biomedical studies, simulation training, human sample studies, drug discovery and delivery studies, biomarker discovery, clinical trials, practice guidelines and its implementation, health care reform, and precision medicine[4,5,13–15].

Translational medicine is a two way process[16] which requires a multidisciplinary team with very close collaborations among scientists, clinicians, policy makers, funding agencies, patent attorneys, technology transfer experts, entrepreneurs, and various industry partners to expedite the translation from bench to bedside and from bedside to bench in order to ultimately provide improved health care to patients with optimal outcome[17–20].

## References

[1] Liu R (2014). The launch of the Translational Perioperative and Pain Medicine. Transl Perioper Pain Med.

[2] Mehic B (2011). Translational research in medicine. Bosn J Basic Med Sci.

[3] Liu JP (2013). A turning point: focusing on translational medicine. Clin Exp Pharmacol Physiol.

[4] Shi L, Lopez Villar E, Chen C (2014). Translational medicine as a new clinical tool and application which improves metabolic diseases: perspectives from 2012 Sino-American symposium on clinical and translational medicine. Clin Transl Med.

[5] Marquet P, Longeray PH, Barlesi F, Ameye V, participants of round table NdoG-XXX (2015). Translational Research: Precision Medicine, Personalized Medicine, Targeted Therapies: Marketing or Science?. Therapie.

[6] Zhang J (2012). Translational medicine in China. Sci China Life Sci.

[7] Parks L (2013). Partnership opens Translational Medicine Center in Suzhou. Bioanalysis.

[8] Cyranoski D (2014). China opens translational medicine centre in Shanghai. Nature.

[9] Geraghty J (1996). Adenomatous polyposis coli and translational medicine. Lancet.

[10] Liang MH (2003). Translational research: getting the word and the meaning right. Arthritis Rheum.

[11] Mayer L (2002). The real meaning of translational research. Gastroenterology.

[12] Zhou J, Wu D, Liu X, Yuan S, Yang X (2012). Translational medicine as a permanent glue and force of clinical medicine and public health: perspectives (1) from 2012 Sino-American symposium on clinical and translational medicine. Clin Transl Med.

[13] Waldman SA, Terzic A (2009). Translational medicine in the era of health care reform. Clin Transl Sci.

[14] Naidu MU (2011). Promise of translational medicine: An evidence-based therapeutics. Indian J Pharmacol.

[15] Nielsen J (2012). Translational and systems medicine. J Intern Med.

[16] Marincola FM (2003). Translational Medicine: A two–way road. J Transl Med.

[17] Ringborg U, de Valeriola D, van Harten W, Bosch AL, Lombardo C (2008). Improvement of European translational cancer research. Collaboration between comprehensive cancer centers. Tumori.

[18] Weston CM, Bass EB, Ford DE, Segal JB (2010). Faculty involvement in translational research and interdisciplinary collaboration at a US academic medical center. J Investig Med.

[19] Splendiani A, Gundel M, Austyn JM, Cavalieri D, Scognamiglio C (2011). Knowledge sharing and collaboration in translational research, and the DC-THERA Directory. Brief Bioinform.

[20] Long JC, Cunningham FC, Carswell P, Braithwaite J (2014). Patterns of collaboration in complex networks: the example of a translational research network. BMC Health Serv Res.

